# The Effect of the Oleophobicity Deterioration of a Membrane Surface on Its Rejection Capacity: A Computational Fluid Dynamics Study

**DOI:** 10.3390/membranes11040253

**Published:** 2021-03-31

**Authors:** Amgad Salama, Adel Alyan, Mohamed El Amin, Shuyu Sun, Tao Zhang, Mohamed Zoubeik

**Affiliations:** 1 Process System Engineering, University of Regina, Regina, SK S4S 0A2, Canada; hmo237@hotmail.com; 2 Reactors Depart., Nuclear Research Center, Atomic Energy Authority, Cairo 13759, Egypt; adelalyan@yahoo.com; 3 Energy Research Lab., College of Engineering, Effat University, Jeddah 22332, Saudi Arabia; mohamed.elamin.kaust@gmail.com; 4 Physical Science and Engineering Division, King Abdullah University of Science and Technology, Thuwal 23955, Saudi Arabia; shuyu.sun@kaust.edu.sa (S.S.); tao.zhang.1@kaust.edu.sa (T.Z.); 5 Mechanical Engineering Department, University of Tripoli, Tripoli 13275, Libya

**Keywords:** oily water filtration, membrane technology, contact angle, multicontinuum approach, CFD

## Abstract

In this work, the effects of the deteriorating affinity-related properties of membranes due to leaching and erosion on their rejection capacity were studied via computational fluid dynamics (CFD). The function of affinity-enhancing agents is to modify the wettability state of the surface of a membrane for dispersed droplets. The wettability conditions can be identified by the contact angle a droplet makes with the surface of the membrane upon pinning. For the filtration of fluid emulsions, it is generally required that the surface of the membrane is nonwetting for the dispersed droplets such that the interfaces that are formed at the pore openings provide the membrane with a criterion for the rejection of dispersals. Since materials that make up the membrane do not necessarily possess the required affinity, it is customary to change it by adding affinity-enhancing agents to the base material forming the membrane. The bonding and stability of these materials can be compromised during the lifespan of a membrane due to leaching and erosion (in crossflow filtration), leading to a deterioration of the rejection capacity of the membrane. In order to investigate how a decrease in the contact angle can lead to the permeation of droplets that would otherwise get rejected, a CFD study was conducted. In the CFD study, a droplet was released in a crossflow field that involved a pore opening and the contact angle was considered to decrease with time as a consequence of the leaching of affinity-enhancing agents. The CFD analysis revealed that the decrease in the contact angle resulted in the droplet spreading over the surface more. Furthermore, the interface that was formed at the entrance of the pore opening flattened as the contact angle decreased, leading the interface to advance more inside the pore. The droplet continued to pass over the pore opening until the contact angle reached a certain value, at which point, the droplet became pinned at the pore opening.

## 1. Introduction

**Figure 1:**
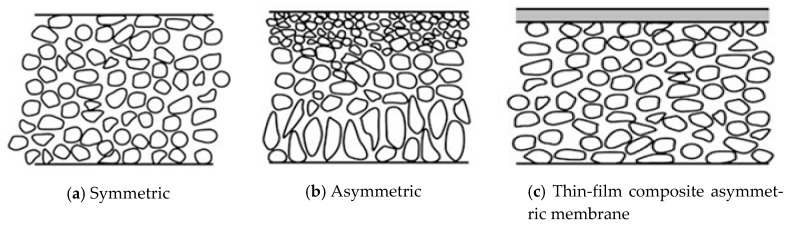
Symmetric and asymmetric porous membranes. In symmetric membranes, the pore structure is, to a large extent, homogeneous, whereas it is not for asymmetric membranes.

Membranes used in separation and filtration processes are manufactured to hold dispersed materials and let a continuous phase pass through them. In general, membranes can be fabricated and conditioned by interfacial polymerization [1,2,3], layer-by-layer assembly [4,5,6], surface grafting, and physical coatings [7,8,9,10]. The two main types of membranes that are widely used in industry are polymeric- and ceramic-type membranes. Polymeric membranes are categorized into different kinds according to the manufacturing process used to make them. However, they can be broadly categorized into two main types, namely, symmetric and asymmetric membranes. A symmetrical membrane has a similar structural morphology at all locations. An asymmetric membrane is made of two or more structural planes of nonidentical morphologies, where each plane has different structures and hydraulic resistances. A typical asymmetric membrane has a relatively dense, extremely thin surface skin layer that is supported on an open, much thicker porous substructure. Figure 1 shows schematics of symmetric and asymmetric porous membranes.

Membranes fabricated via phase inversion are generally selected as the substrates, which provide mechanical support. Phase inversion is defined as a process of controlled polymer transformation from a liquid phase to a solid phase and is a common method that is used in the fabrication of membranes. In this method, solvents are removed from the liquid–polymer solution, leaving a porous, solid membrane. The method of phase inversion is highly dependent on the type of polymer used and on the solvent that is used to dissolve the polymer. Thin-film composite membranes consist of a thin dense film of highly cross-linked polymer that is formed on the surface of a thicker microporous support. 

**Figure 2:**
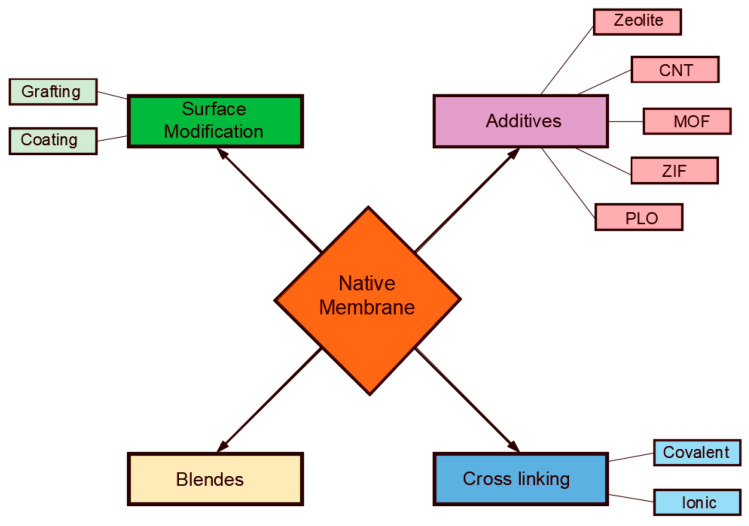
Schematic diagram of the different techniques that are used to modify the surface properties of membranes. CNT: carbon nanotube, MOF: metal–organic framework, ZIF: zeolitic imidazolate frameworks, PLO: porous layer oxides (Reproduced with permission from [12]. Wiley, 2013.)

The porous structure of the membrane is the key factor that determines its selectivity features. The sizes of the pore openings are chosen such that they can achieve the required purification level. In general, they must be smaller than the size of the dispersed materials such that they can hold them at the surface. Since the pore openings of membranes present a distribution of sizes, and likewise, dispersed materials present a distribution of sizes, there are occasions in which small dispersals land over large pore openings, and in such cases, permeation occurs. Dispersed materials in fluids can be of two types, namely, solid particles or droplets of other immiscible fluids. When the dispersed materials are droplets of immiscible fluids within the continuous phase, separation would also depend on the surface affinity properties of the membrane toward the dispersed droplets. That is, fluids are generally prone to deformations that enable them to flow with the continuous fluid unless interfacial forces are strong enough to keep them intact. Interfacial forces depend on the surface tension and the contact angle at the solid surface. It is required that dispersed droplets form interfaces at pore openings to prevent them from permeating. Such interfaces must be concave toward the pinning droplets to achieve a holding capacity against permeation. This would be the case when the contact angle along the contact line is greater than 90°. In other words, the surface of the membrane must be conditioned such that it is non-wetting for the dispersed droplets. The larger the contact angle, the harder it will be for the pinned droplets to permeate through the membrane and vice versa. Such a nonwetting character of the membrane surface for the pinned droplets may be natural or artificial. In most cases, the surface of membranes may need to be treated to achieve sufficient nonwetting capabilities. The results of the modification will yield different membrane responses from pinned droplets, fouling development, and absorption characteristics [11]. Several methods have been proposed for surface conditioning, including coatings, chemical grafting, polymer blends, plasma, and surfactants. Figure 2 shows a schematic of the different methods that are used to modify the surface properties of the membrane. Coating materials for application in membrane technology have been developed to acquire desired affinity properties (in terms of hydrophilicity, oleophobicity, etc.). Coating refers to the application of a thin-film layer of a different polymer or monomer system to form a new surface via composite formation (e.g., through polymerization processes).

To list some advances in this regard, Shao et al. [13] suggested the self-polymerization of polydopamine, which acts as a glue for the entrapment of hydrophilic TiO_2_ nanoparticles. They reported an increase in the hydrophilicity characteristics of the membrane and the permeation flux. Similarly, Cheng et al. [14] developed a surface modification of PES (polyether sulfone) membranes via the interfacial polymerization of amino-functional polyethylene glycol (PEG) and trimesoyl chloride. The higher hydrophilic moiety, the positive surface charge, and the larger pore size were the main factors contributing to the improved performance of the membrane. Galiano et al. [15] introduced a PBM (polymerized bicontinuous microemulsion), which was prepared, polymerized, and used as a coating material for the surface modification of commercially available PES membranes [16]. Chemical grafting, on the other hand, is the process of attaching a low-molecular-weight active group (monomer) to a parent polymer or membrane. Surface grafting is one of the most promising methods for modifying the membrane surface through the covalent bonding interaction between the grafted chains and the membrane [17,18]. Some membranes exhibit low reactivity (e.g., polyvinylidene fluoride (PVDF)), which makes it difficult for biomolecules to couple with its surface in a covalent manner; thus, for this aim, the grafting of functional groups is required [19,20,21,22]. On the other hand, metal–organic frameworks (MOFs) have recently been introduced to the synthesis of functional porous materials, where the hydrophobicity can be controlled or tuned. The existence of inorganic and organic species that are arranged in a regular pattern provide countless numbers of MOFs that can be synthesized using different metal and organic linkers [23,24]. Metals or metal oxides represent the inorganic component in MOFs and they occupy the nodes, while the organic part is a multidentate ligand, such as benzene carboxylic acid. Moreover, thin films of MOFs were shown to be effective at tuning membranes’ affinities for water and wastewater treatment [25,26]. 

Another method of changing the affinity properties of the membrane includes the use of polymer blends (PBs). PBs are mixtures of two or more different polymers that can improve the chemical properties of membranes and their separation performance. It is to be noted that permanent surface modification by grafting hydrophilic groups and crosslinking coatings are strategies that are attempted to reduce the problem of fouling. However, changes in the membrane structure and integrity often result. Recently, several studies have explored the passive adsorption of surfactants and soluble polymers as an economical means of achieving similar membrane modification. The use of surfactants refers to treatment with water-insoluble material to enhance the hydrophilicity (i.e., improve wetting) [27,28,29,30,31]. However, it has a potential problem related to the possibility that produced water may get contaminated due to leaching. This can also cause deterioration of the selectivity features of membranes, particularly those used in the filtration of oily water systems. In other words, the leaching of affinity-enhancing surfactants with time can lead to the deterioration of the contact angle, which in turn can lead to the premature permeation of pinned droplets that would otherwise not permeate. This will happen particularly when the crossflow filtration methodology is used. In this case, the flow of the feed stream along the membrane surface continuously erodes and washes any affinity-modifying agents from the surface. The continuous leaching of affinity-enhancing agents at the membrane surface will decrease the rejection capacity of the membrane and cause a significant loss of functionality [32]. 

Produced water is a class of wastewater that is usually associated with the production of oil. Water is used to displace the oil and, in the process, it carries the oil with it to the production facilities. At the wellhead, the majority of the oil is separated from the water using physical methods. Even though the leftover water contains less oil, it cannot be disposed of directly; it needs to go for further separation before it can be disposed of. One of the effective methodologies that have been used in the filtration of produced water is the use of membrane technology. The selectivity feature of membranes when filtering produced water depends not only on their pore sizes but also on the interfacial forces. Therefore, membrane surfaces are usually treated such that they exhibit a nonwetting character for the dispersed phase. As introduced earlier, the processes of enhancing the affinity properties of membranes include surface grafting or the addition of surface coatings, surfactants, polymer blends, etc. However, concerns remain about the persistence of affinity-enhancing agents and the associated wearing or leaching, particularly in crossflow filtration. This can lead to the deterioration of membrane functionality with time. In this work, we explored this topic and showed how the deterioration of the contact angle (as a manifestation of the wettability state of the membrane) can influence its performance. A method was introduced to quantify the loss of performance of a membrane due to the deterioration of its affinity-related characteristics. The study was conducted via the tools of computational fluid dynamics (CFD) at the microfiltration scale. It aimed at studying the behavior of an oil droplet along the membrane surface as the contact angle progressively decreased. Since the contact angle influences both the critical entry pressure and the critical velocity of dislodgment, it was expected that the fates of the droplets at the membrane surface would change with a decrease in the contact angle. In the next two sections, we provide details on how the contact angle influences the critical conditions. We also provide an expression for the critical entry pressure for the case in which the wettability conditions are different at the surface and inside the pores.

## 2. On the Fate of Oil Droplets at a Membrane Surface

**Figure 3:**
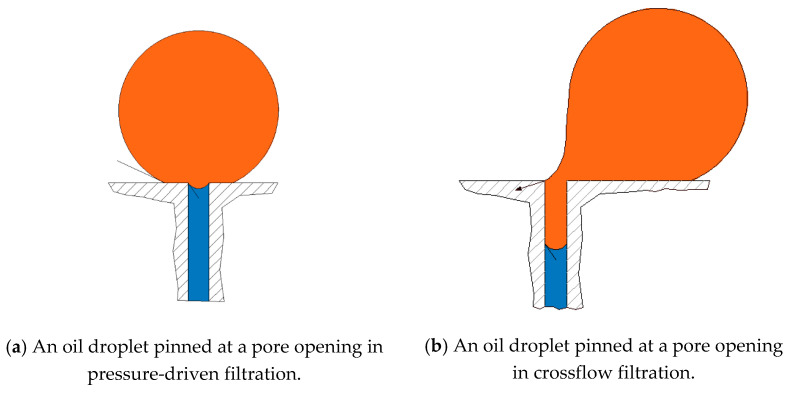
When an oil droplet is pinned over a pore opening, it can assume a symmetrical profile (**a**), as is the case for pressure-driven filtration, or a deformed profile (**b**), as in the case for crossflow filtration, with the degree of deformation being dependent on the crossflow velocity.

An oil droplet that is pinned at a pore opening forms an interface at the inlet of the pore that prevents its permeation through the membrane unless the pressure is large enough. For every droplet and pore opening size, there exists a threshold entry pressure above which the pinned droplet permeates. An estimation of the entry pressure was investigated by Nazzal and Wiesner [33] and Salama [34,35] for the cases when the droplets were assumed to be spherical, which is an assumption that is sufficiently accurate for the sizes of droplets and pore openings that are typically found in produced water filtration. In a class of separation processes known as crossflow filtration, the feed stream flows along the membrane length. The shearing force generated by the flow field deforms the droplets and can cause variations in the entry pressure magnitudes. However, for the purpose of analysis, it has been suggested by several authors [36,37,38] that the formula derived for the entry pressure when assuming approximately spherical droplets may still be valid for the analysis of the filtration process of typical produced-water systems. Figure 3 shows a droplet undergoing pinning for two cases, namely, (a) pressure-controlled filtration and (b) crossflow filtration. Four fates have been identified for oil droplets when they are pinned on a membrane surface in crossflow filtration. These fates are determined based on the relationship between two operating and two critical conditions. The two operating conditions include the transmembrane pressure (TMP) and crossflow velocity (CFV), and the two critical conditions include the critical entry pressure $p_{crit}$ and the critical velocity of dislodgment $v_{crit}$. When both the TMP and the CFV are larger than the critical conditions, the oil droplets tend to breakup (i.e., part of the droplet permeates and the other part stays on the feed side). When the TMP is larger than the critical entry pressure, while the CFV is smaller than the critical velocity, the pinned droplet permeates. When the TMP is smaller than the entry pressure, no permeation occurs and the droplet can either stay pinned or move along the surface according to the crossflow velocity. A fate map for every size of oil droplets in relation to all sizes of porous membranes may be constructed where both the operating and critical conditions define areas where the four previously mentioned fates are located. Salama et al. [37,38,39] used this map to build a multicontinuum framework that describes the filtration process of an oily water system and determines the rejection capacity of the membrane. In the multicontinuum approach, both the droplet and pore size distributions are divided into several size ranges, with each size range defined as a continuum. The rules that determine the fate of each oil continuum for all membrane continua are essentially based on comparing the operating conditions with the critical conditions. Such a comparison determines the location in the fate map of each oil continuum and, therefore, suggests its rejection potential. This technique was used in this study to show how the deterioration of the contact angle can shift the rejection capacity of the membrane toward the incoming flux of oil droplets.

## 3. On the Critical Conditions

### 3.1. Critical Entry Pressure

**Figure 4:**
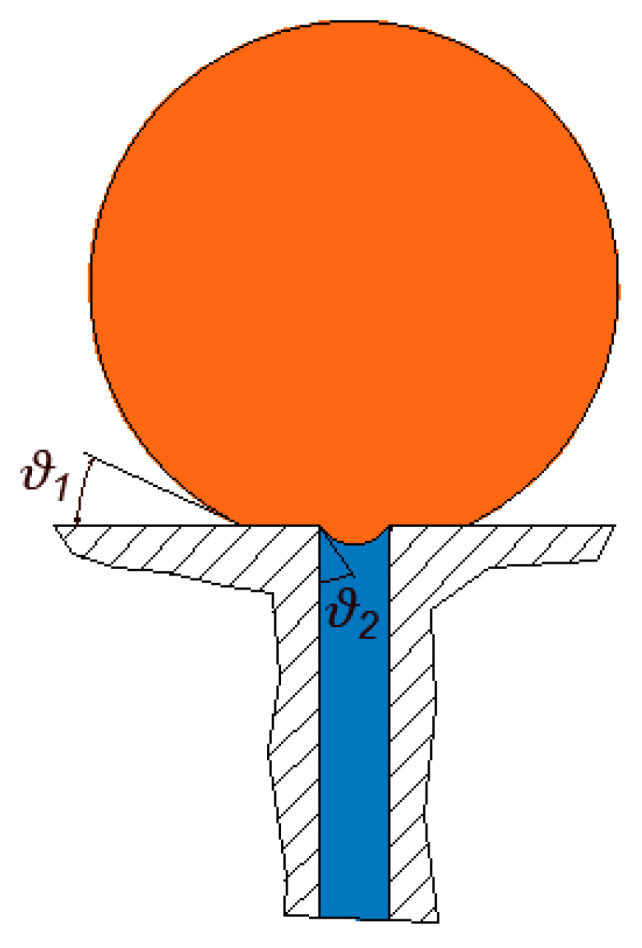
The wettability conditions at the surface of the membrane and inside the pores may be different. In such a case, the contact angles that the two interfaces make are different.

**Figure 5:**
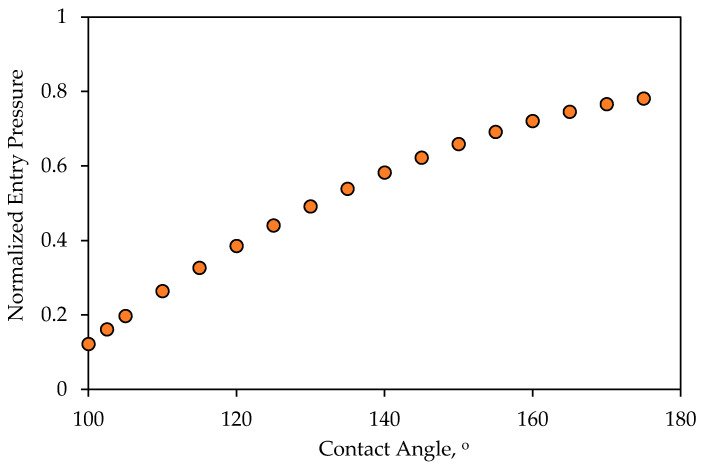
Normalized entry pressure variations with the contact angle (which was assumed to be the same at the surface of the membrane and inside the pore) for the case in which $r_{d}=40\;\mu m$,$\;r_{p}=2\;\mu m$, and σ=0.035 N/m. The pressure value was normalized relative to the capillary pressure of the interface inside the pore, i.e.,$\;2\sigma \cos\vartheta_{2}/r_{p}$.

Since the selectivity feature of the membranes used in the filtration of oily water systems is very much related to interfacial phenomena, it is important that a clear understanding of its role is established. When a droplet is pinned over a pore opening, an interface is formed that can prevent the movement of the droplet through the pore opening unless the TMP exceeds a threshold value called the entry pressure. Therefore, if the operating pressure is kept smaller than the entry pressure, the droplet will essentially stay pinned at the surface of the membrane. The contact angle is an important parameter for determining the entry pressure. When the contact angle is reduced (for example, due to the leaching of affinity-enhancing agents), the threshold entry pressure also decreases. In other words, the droplet would require less pressure to start permeating through the membrane when the contact angle is decreased. It is interesting to note that the application of affinity-enhancing agents may not soak well underneath the surface layer in some cases. In other words, it is possible that the wettability conditions of the membrane are not uniform such that the contact angle at the surface of the membrane is different than it is inside the membrane pores. In this case, the interface(s) formed at the pore openings may construct different contact angles than it would at the surface. The formula that was developed by Nazzal and Wisner [33] to estimate the critical pressure only accounts for those cases in which both interfaces (i.e., at the surface of the membrane and inside the pore opening) are assumed to have the same contact angle. In order to account for the case in which the contact angles may be different, in this work, we generalized the formula developed in [33]. To do so, let the contact angle of the interface at the surface of the membrane be $\vartheta_{1}$ and that at the pore openings be $\vartheta_{2}$; it is easy to show that the critical entry pressure formula developed by Nazzal and Weisner [33] may be modified to give the following:

(1) $$P_{crit}=\frac{2\sigma \cos\vartheta_{2}}{r_{p}}\left(1-\sqrt[{3}]{\frac{2+3\cos\vartheta_{1}-\cos^{3}\vartheta_{1}}{4{\left(\frac{r_{d}}{r_{p}}\right)}^{3}\cos^{3}\vartheta_{2}-\left(2-3\sin\vartheta_{2}+\sin^{3}\vartheta_{2}\right)}}\right),$$

where $P_{crit}$ is the entry pressure, σ is the interfacial tension, $r_{p}$ is the radius of the pore opening, $r_{d}$ is the radius of the droplet, $\vartheta_{1}$ is the supplement to the contact angle (contact angle = $180^{{}^{\circ}}-\vartheta_{1})$ at the surface of the membrane and, likewise, $\vartheta_{2}$ is the supplement to the contact angle inside the pore, as shown in Figure 4. It is to be noted that this formula reduces to that of Nazzal and Wisner [33] in the case when $\vartheta_{1}=\vartheta_{2}$. In the Appendix A, a derivation of Equation (1) is provided. According to Equation (1), the critical entry pressure is a function of many factors, namely, the contact angles, the diameter of the droplet, the diameter of the pore opening, and the surface tension. Figure 5 shows the variations of the entry pressure with the contact angle. According to Figure 5, the critical entry pressure is smaller for smaller contact angles. 

**Figure 6:**
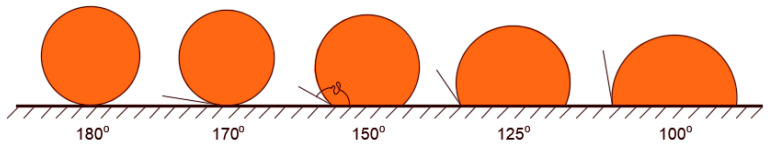
Succession of configurations of oil droplets at the surface of a membrane with different contact angles. When the contact angle is smaller, the droplet tends to spread over the surface more.

As has already been highlighted, in crossflow filtration, the flow field can result in the leach and washout of the affinity-enhancing agents, which may have been added during the manufacturing process to treat and enhance the performance of the membranes. The washout of these materials gradually deteriorates the rejection capacity of the membrane. In other words, the contact angle continuously decreases with time, therefore increasing the chance of the permeation of oil droplets and reducing the rejection capacity of the membrane. This process renders the membrane obsolete and causes a loss of capital and increases running costs. Figure 6 schematically shows successive configurations of an oil droplet at the surface of the membrane when the contact angle decreases. As shown, the droplet spreads over the surface more when the contact angle is smaller.

### 3.2. Critical Velocity of Dislodgment

**Figure 7:**
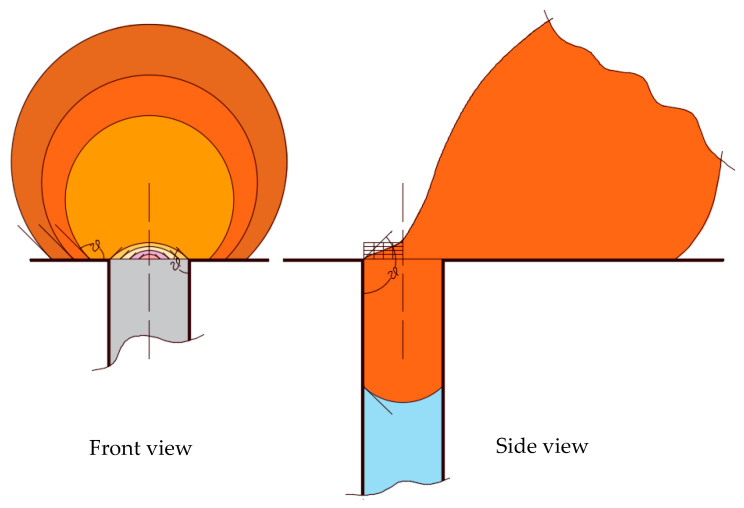
The critical configuration is achieved when the contact angle of the receding interface assumes the static contact angle. Any further deformation will lead to the breakup of the droplet. The front view shows the projection of the droplet at the frontal cross-section and the side view depicts the deformation of the droplet due to the crossflow field.

The decrease in the contact angle not only affects the magnitude of the entry pressure but also the magnitude of the critical velocity of dislodgment. When the droplet spreads over the surface more, it requires a larger velocity for its dislodgement. Figure 7 shows a schematic of the critical configuration of a pinned droplet in crossflow filtration. The critical configuration is defined as that configuration at which the droplet breaks up into two portions: one in the pore and another in the feed. As the receding part of the interface reaches the pore opening, it gets anchored and starts to deform. The deformation continues until the interface assumes the static contact angle, as shown in Figure 7. 

**Figure 8:**
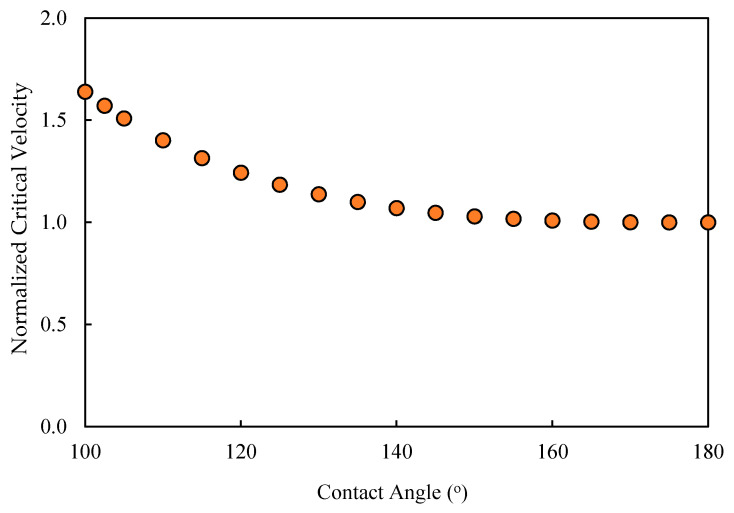
Normalized critical velocity of dislodgment variations as a function of the contact angle. This figure shows that the critical velocity of dislodgment decreases with the increase in the contact angle, which was assumed to be the same at the surface of the membrane and inside the pore. The critical velocity was normalized relative to the critical velocity when the contact angle is 180°.

At this instant, any further deformation will lead to the breakup of the droplet. Salama [36] recently developed a formula for calculating the critical velocity of dislodgment in crossflow filtration (in the case of laminar flow conditions in the feed channel). It takes the following form:

(2) $$v_{avg}=\frac{1}{108\varepsilon \left(\theta \right)}\left(\frac{H}{r_{d}}\right){\left(\frac{r_{P}}{r_{d}}\right)}^{2}\left[\frac{\left(4+3\pi \right)\sigma }{\zeta \mu_{w}}\right]\;,$$

where *H* is the height of the feed channel, $r_{d}$ is the radius of the droplet, $r_{P}$ is the radius of the pore opening, σ is interfacial tension, $\mu_{w}$ is the viscosity of the continuous phase, θ is the supplement of the contact angle, ε(θ) is a geometrical factor for the cross-sectional area of the droplet, and ζ is a coefficient that accounts for the spread of the droplet over the surface (≈cosθ, where θ is the supplement of the contact angle). This velocity represents the crossflow velocity that is required to dislodge permeating droplets. Figure 8 shows the variations of the critical velocity of dislodgment and the contact angle, which indicates that the critical velocity of dislodgment increases with the degradation of the contact angle. The critical velocity of dislodgment also depends on the viscosity contrast between the droplet and the continuous fluid, Salama [40,41]. It also determines the volume of the droplet at breakup inside the pore and at the surface, Salama [42]. 

## 4. CFD Investigation

The flow and transport of multiphase systems are complicated due to the existence of interfaces that are deformable and undergo general three-dimensional motion. Researchers in this field have adapted several frameworks that depend on the categories of the multiphase systems. Different patterns of multiphase systems exist according to the distribution of the phases in the bulk continuous phase, usually via visual observation. It can, however, be difficult to specify with certainty which regime a particular flow belongs to. Since researchers may not agree on a unique set of flow regimes, many other classifications exist in the literature. Classification can be useful since different flow regimes affect parameters in different ways, such as a pressure drop. For two-phase systems, flow regimes determine the macroscopic behavior of the system, as well as the appropriate modeling approaches. The flow regimes can be divided into three main classes, namely, (i) regimes for horizontal flow in channels, where gravity tends to locate the heavier phase closer to the bottom; (ii) regimes for vertical flow in pipes, where the liquid phase tends to be on the pipe walls, forming a stable or an unstable film; (iii) regimes for sloped pipes, in which the slope angle is important, as well as the direction of the flow (upward or downward). The general flow regimes can be stated as bubbly flow, plug flow, stratified flow, wavy flow, slug flow, churn flow, annular flow, etc. In produced water applications, the dispersed phase represents tiny oil droplets that resemble the bubbly flow regime. Models related to the flow of multiphase systems are very much dependent on the type of flow regime. They can generally be classified into continuum-type approaches, lumped parameter approaches, or interface-tracking approaches (e.g., volume of fluid (VOF) method [43], level-set method [44], diffuse interface model [45,46], and lattice Boltzmann methods [47]). In this research, since we were interested in the fate of oil droplets at the surface of the membrane under oleophobicity deterioration, interface tracking methods were used. In particular, the VOF method as adapted in ANSYS Fluent R19 [48] was used to study this problem because of the advantage that it is locally conservative. 

### 4.1. Governing Equations

The governing equations that modeled this system were those representing the conservation of mass and momentum, in addition to constitutive relationships, to calculate the properties in the interface region. An equation for the advection of a phase function was used to track the interface and to calculate the interfacial tension forces. This binary function was defined such that it is one in one phase and zero in the other phase. The interface was defined along the cells where the phase-field function was assumed to have a value between 0 and 1. The governing equations describing this system based on the VOF model include:

Continuity:

(3) ∇·ρv=0,

where v is the velocity vector and ρ is the density of the combined system.

Momentum:

(4) $$\frac{\partial \rho v}{\partial t}+\nabla \cdot \rho vv=-\nabla p+\nabla \cdot \mu \left(\nabla v+\nabla v^{T}\right)+\rho g+F_{\sigma },$$

where g is the acceleration due to gravity and $F_{\sigma }$ is the surface tension force per unit volume.

Transport of phase-field function:

(5) $$\frac{\partial \varphi }{\partial t}+\nabla \cdot \varphi v=0,$$

where φ is a binary phase-field function that defines the phases.

Constitutive relationships:

These relationships were developed to determine the properties based on the volumetric contribution of each phase in each cell:

(6) $$\rho =\varphi \rho_{1}+\left(1-\varphi \right)\rho_{2},$$

where $\rho_{1}$ and $\rho_{2}$ are the densities of the two phases.

(7) $$\mu =\varphi \mu_{1}+\left(1-\varphi \right)\mu_{2},$$

where $\mu_{1}$ and $\mu_{2}$ are the viscosities of the two phases. The interfacial tension force per unit volume was modeled using:

(8) $$F_{\sigma }=\sigma \frac{\rho \kappa \nabla \varphi }{\frac{\rho_{1}+\rho_{2}}{2}},$$

where σ is the interfacial tension and κ is the curvature of the interface. The normal unit vector at the interface was calculated using:

(9) $$n=\frac{\nabla \varphi }{\left|\nabla \varphi \right|}.$$

**Figure 9:**
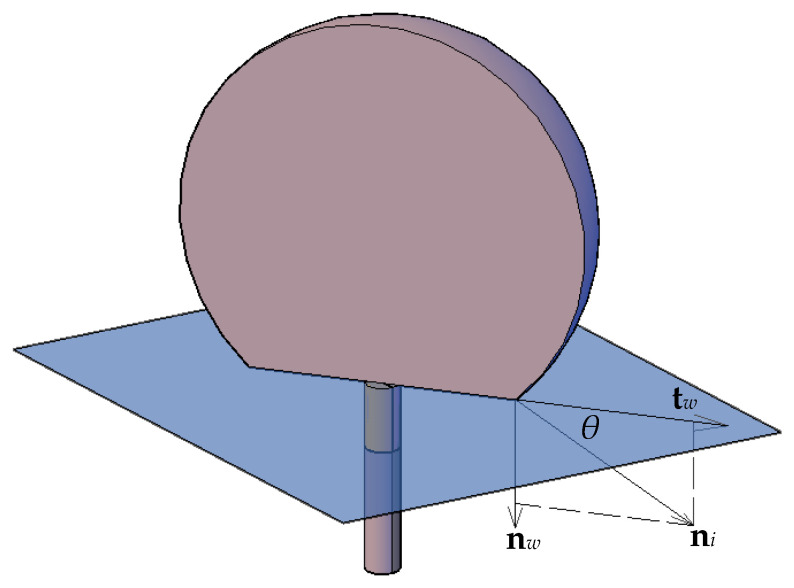
A 3D representation of the interface with the unit normal vector in the cells at the walls along the contact line.

To adjust the surface normal in cells near the wall, the so-called dynamic boundary condition developed by Brackbill et al. [49] was used. Therefore, at the contact line, the normal unit vector may be resolved in the plane that is normal to the contact line as:

(10) $$n_{i}=\cos\vartheta_{s}n_{w}+\sin\vartheta_{s}t_{w},$$

where $n_{i}$ is the normal unit vector at the contact line, $n_{w}$ and $t_{w}$ are the unit vectors normal and tangential to the wall, respectively, and $\vartheta_{s}$ is the static contact angle. The combination of this contact angle with the normally calculated surface normal one cell away from the wall determines the local curvature of the surface, and this curvature is used to adjust the body force term in the surface tension calculation. Figure 9 shows a schematic 3D pictorial view of the interface and the normal and tangential vectors at the surface with the contact angle. The curvature $\kappa \left(x_{i}\right)$ was determined using:

(11) $$\kappa =\frac{1}{\left|n\right|}\left[\left(\frac{n}{\left|n\right|}\cdot \nabla \right)\left|n\right|-\left(\nabla \cdot n\right)\right].$$

These equations describe a nonlinear system that requires numerical techniques to acquire solutions. Several numerical methods are available but the finite volume method was chosen in this work, as described in the next section.

### 4.2. The Numerical Algorithm

**Figure 10:**
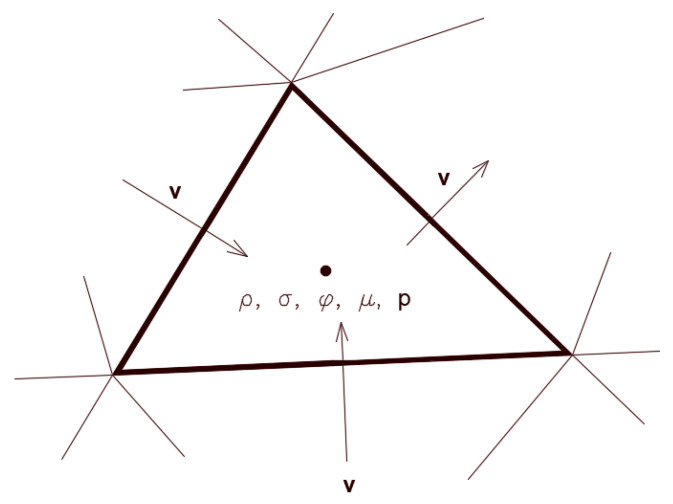
Schematic of a typical finite volume element over which the governing equations were discretized. In the finite volume method, variables are defined at either cell centers or cell faces. Usually, the velocity and stress tensors are defined at the faces, while fluid properties, pressure, and the phase-field function are defined at cell centers.

The system of equations that describes the conservation principles as given before are partial differential equations that are applicable at every point of the computational domain. Another way of representing these equations can be through integration with respect to the elemental volume. In the integral form, terms that are volume-related are integrated over the volume and terms that reflect the transport across the surfaces are integrated over the area. As an example, the momentum equation can be integrated over the volume as follows:

(12) $$\int_{v}\left(\frac{\partial \rho v}{\partial t}+\nabla \cdot \rho vv-\nabla \cdot \sigma -\rho g-F_{\sigma }\right)dv=0,$$

where σ is the stress tensor. Using the divergence theorem, the above equation reduces to the following equation, which involves both volume and surface integrals:

(13) $$\frac{\partial }{\partial t}\int_{v}\rho vdv+\int_{A}\rho vv\cdot ndA-\int_{A}\sigma ndA-\int_{v}\left(\rho g+F_{\sigma }\right)dv=0,$$

where n is the outward normal vector. The above equation involves integration with respect to elemental volume and its surface areas. Therefore, in the numerical algorithm, some variables are defined at the cell center and some other variables are defined at the faces, as shown schematically in Figure 10. An operator splitting scheme was used to solve the coupled system. In this approach, the time-dependent Navier–Stokes equations, together with the continuity equation, were solved to update the velocity field, assuming the values of the surface tension force from the previous time step. In other words, the configuration of the droplet from the previous time step was used to calculate the surface forces that were used in the balance equations to update the velocity. The new velocity field was then used to update the configuration of the droplet and the properties of the mixture. The solution was obtained iteratively at each time step until the predefined tolerance was obtained (a tolerance of 10^−4^ was considered for all variables). An adaptive time step that ranged between 10^−6^ and 10^−8^ s was used to accelerate the solution. In this study, we considered a region in space that represented the feed channel in the form of a rectangular parallelepiped. In the middle of the bottom wall, a single pore opening was constructed downward to resemble a pore opening.

**Figure 11:**
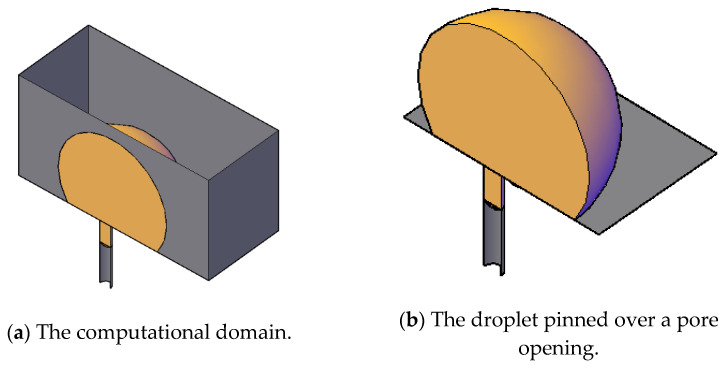
Pictorial schematic view of the computational domain (**a**) (note that the top face has been removed to show the droplet) and of the droplet (**b**) upon permeation through the pore opening.

The dimensions of the domain spanned a length of 10 microns, a width of 7 microns, and a height of 2.1 microns. In the middle of the bottom face of the rectangular prism, a hole of diameter 4 microns with an extended downward length of 1.5 microns was made to present a cylindrical pore. Figure 11 shows a pictorial view of the schematic 3D computational domain and a closer view of the droplet over the surface and the pore opening. This setup was similar to our previous work, Zoubeik et al. [50] and Salama [40,41,42], as well as the work of other researchers. Plenty of validation exercises can be found in our previous works [50]. In this setup, the domain was discretized into uniform rectangular prisms except closer to the pore opening using ANSYS ICEM. Mesh sensitivity analysis was conducted to reach the optimal mesh that provided an accurate solution and, at the same time, faster convergence. Three mesh resolutions were considered, namely, the base mesh (approximately 1.7 million hexahedral elements), two times denser, and half the mesh size. Both the base mesh and the denser mesh produced similar behavior, and therefore, throughout this study, the base mesh was used. An adaptive time-stepping scheme was used. The maximum time step was restricted to 10^−7^ s. Details about the mesh sensitivity analysis can be found in Zoubeik et al. [50]. Validation of the numerical study was confirmed by reproducing results similar to those found in the literature for similar setups. Again, in Salama et al. [40,41,42] and in Zoubeik et al. [50], comprehensive validation exercises can be found, which built confidence in the modeling approach.

## 5. Results and Discussion of the CFD Study

In the introduction and subsequent sections, discussions on the fates of the oil droplet at the membrane surface were introduced. The critical conditions under crossflow filtration were identified as the critical entry pressure and the critical velocity of dislodgment. These two conditions, along with the two operating conditions, which are the TMP and the CFV, govern the fate of the droplets at the membrane surface. It was indicated that the contact angle appears in both the formulae for the critical entry pressure and the critical velocity of dislodgment. Therefore, it is expected that a change in the contact angle would change the fate of the droplet at the membrane’s surface. Therefore, the purpose of this CFD analysis was to confirm the changes of the fates of the droplet upon the change in the contact angle and to identify whether there may be other unexplored fates. The CFD scenarios considered the following setup: if we start with a droplet at the surface of the membrane for which the TMP is smaller than the critical entry pressure, then any relatively larger CFV will displace the droplet along the membrane surface. If the contact angle decreases (i.e., the surface becomes less nonwetting), then the critical entry pressure will also decreases (Figure 5). If the nonwetting conditions of the surface of the membrane continue to decrease, there will be a time at which the critical entry pressure equalizes with the TMP or even becomes smaller. In such a case, the interface the droplet makes at the entrance of the pore opening starts to advance inside the pore. On the other hand, from the perspective of the critical velocity of dislodgment, the decrease in the contact angle results in an increase in the critical velocity of dislodgment (Figure 8). This implies that the decrease in the contact angle would make the droplet susceptible to permeation and, at the same time, make the CFV unable to dislodge the droplet while permeating. Such an understanding in anticipating the behavior of the oil when the contact angle decreases needs confirmation, which was the focus of the proposed CFD study. In addition, new fates could have emerged that were not anticipated before. In the next paragraph, the CFD setup, along with the operating conditions, is highlighted. The results are shown as snapshots of the droplet at different times with the change in the contact angle.

**Figure A2:**
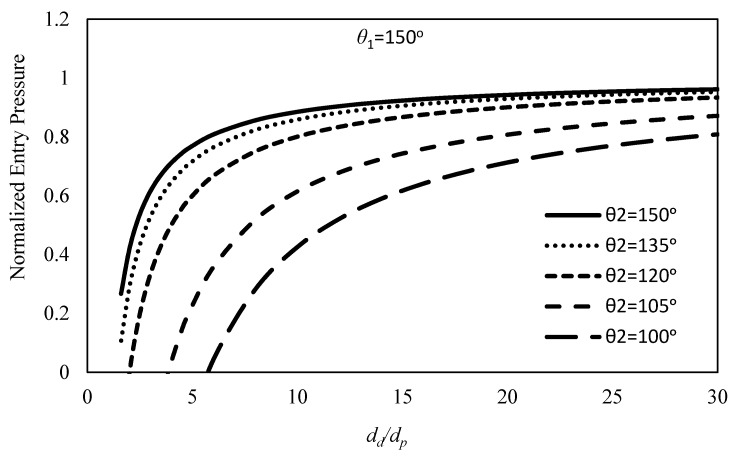
The entry pressure normalized relative to the capillary pressure at the pore entry $\left(4\sigma \cos\theta_{2}/d_{p}\right)$ as plotted against the ratio of the droplet $\left(d_{d}\right)$ versus the pore $\left(d_{p}\right)$ diameters. From this graph, it can be seen that in general, when this ratio is smaller, the entry pressure is also smaller. The contact angle of the interface at the pore entrance has a significant effect, as shown on the graph.

Three scenarios were considered in this study. These scenarios differed in the considered TMP, namely, 0.5, 0.75, and 0.9 bars. The velocity of the top surface of the computational domain in all three scenarios was set to 0.5 m/s. In the first scenario (i.e., TMP = 0.5 bar) the contact angle started at 135° and was reduced in increments of 5°. In the second scenario (i.e., TMP = 0.75 bar), the contact angle was reduced in increments of 2.5°, and in the last scenario (TMP = 0.9 bar), it was reduced in increments of 1°. The simulation results show that, in the first scenario, the droplet continued to be rejected from permeation through the pore until the contact angle was reduced to 105°, when it started to permeate and was no longer rejected. In the second scenario, the droplet became pinned at the pore opening when the contact angle was reduced to 122.5°. In the third scenario, pinning occurred when the contact angle was reduced to 128°. Apparently, earlier permeation of the droplet in the three scenarios could be achieved if the contact angle of the pore was smaller than that at the surface, as discussed earlier. This was attributed to the fact that when the contact angle for the interface that was formed at the entrance of the pore became smaller, an earlier breakthrough was obtained, particularly for smaller droplets. This is manifested in Figure A2 (in the Appendix A), which depicts the entry pressure values for different droplet sizes and different contact angles. From this figure, it is clear that the threshold of oil droplet permeation dropped significantly when the contact angle of the interface at the pore opening was smaller. It was also noted that the influence of the contact angle at the pore entrance was very much controlling the permeation threshold of the pinned droplets more than that at the surface of the membrane, as will be shown later. 

**Figure 12:**
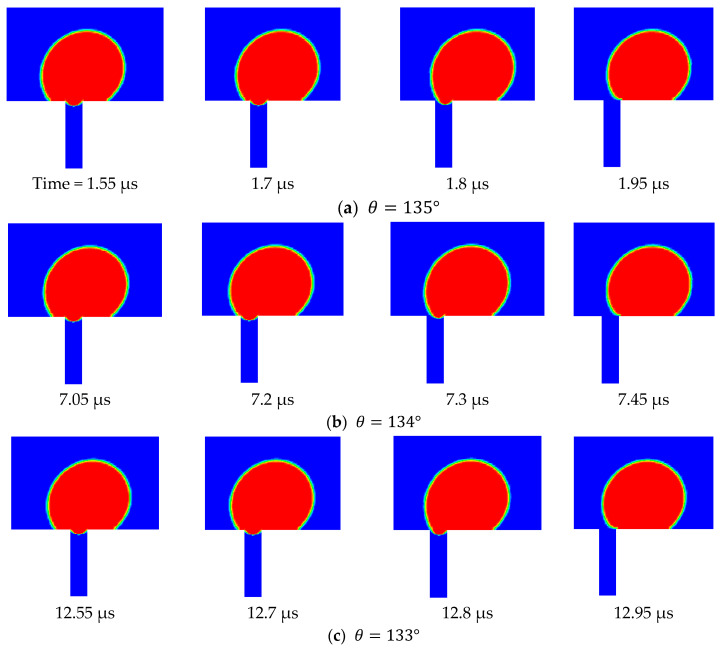

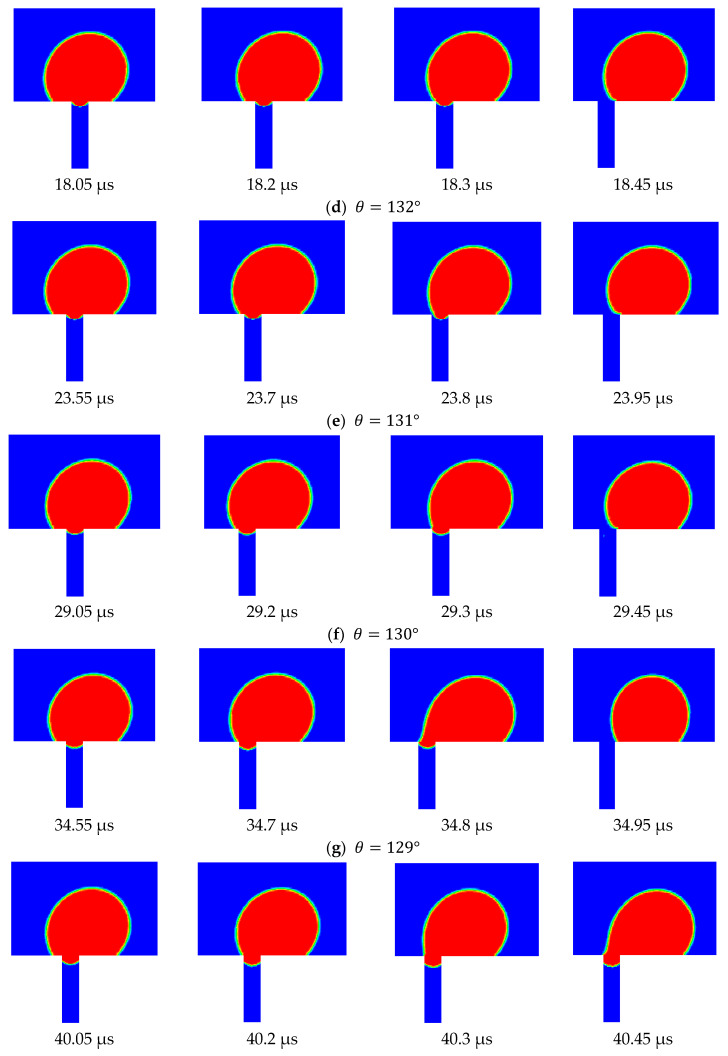

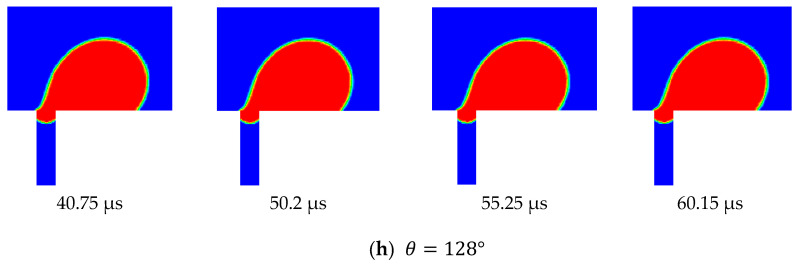
Side view snapshots of the droplet for different contact angles. It is clear that when the contact angle was large and no pinning occurred, the droplet passed along the pore opening and the interface at the pore opening recoiled toward the droplet. As the contact angle became smaller, two things happened, namely, the droplet spread over the surface more and the interface inside the pore moved downward. This continued until the droplet was no longer able to either recoil at the interface inside the pore or it was broken. In the considered example, the following parameters were set to be constant: $\mathrm{TMP}=0.9\;\mathrm{bar},\;v_{top\;wall}=0.5\;m/s,\;\sigma =0.025\;N/m,\;\theta$ varied between 135° and 128°.

Figure 12 shows the side view snapshots of the droplet for the last scenario (i.e., the scenario with TMP equals 0.9 bar) at different times and for different contact angles. From this figure, it is clear that, with the decrease in contact angle, the droplet spread over the surface more. Furthermore, the interface inside the pore opening flattened as the contact angle decreased. This indicates that less pressure would be required to push the droplet inside the pore. Furthermore, with the decrease of the contact angle, the volume of that portion of the droplet that entered the pore opening increased until the case in which the contact angle had become 128°. 

**Figure 13:**
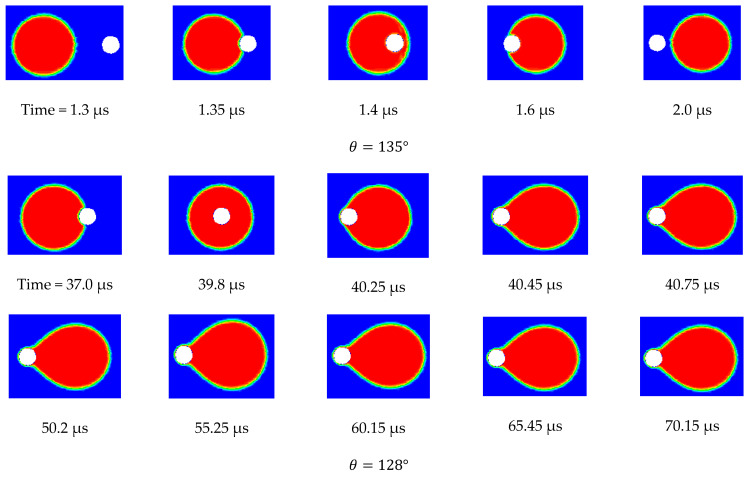
Top view snapshots of the droplet showing that when the contact angle was large and no permeation occurred, the droplet encountered the pore opening and passed through it. The shape of the contact line, in this case, was almost circular. When the contact angle was reduced and pinning conditions occurred, the droplet became held and was no longer able to move. It is also clear that the contact area was larger for smaller contact angles.

Under this condition, the droplet moved along the membrane and the interface at the pore opening moved downward. When the drag force from the crossflow field balanced the surface tension force at part of the pore opening, the droplet stayed pinned at the pore opening. Figure 12 shows the top view snapshots of the droplet for the last scenario. The two cases when the contact angle was 135° and 128° are presented in this figure. It is clear that when no permeation or pinning occurred, the droplet encountered the opening and passed through it while the contact line assumed an almost circular shape. When the contact angle was reduced, the droplet spreads over the surface, covering a larger area. With the continual degradation of the contact angle, the droplet finally becomes captured and is no longer able to move. Under this scenario, the droplet profile at the surface of the membrane was elongated as shown in Figure 13.

**Figure 14:**
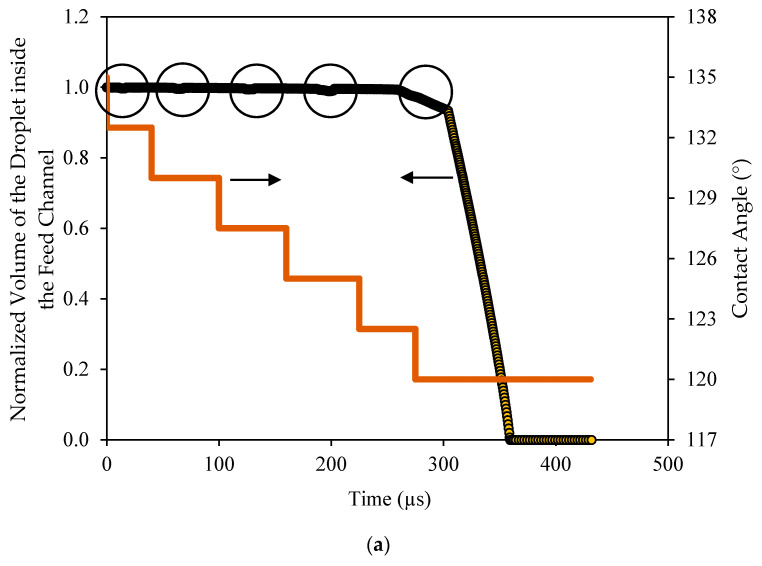

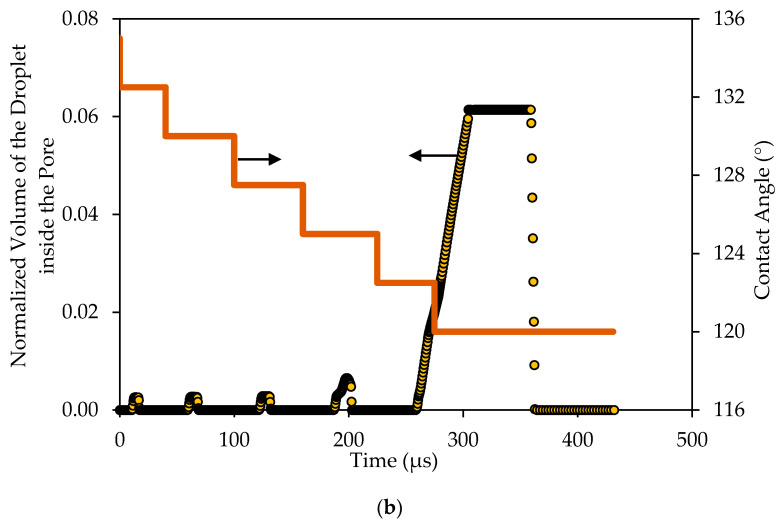
Normalized volume of the droplet (normalized relative to the initial volume of the droplet) in (**a**) the feed channel and (**b**) inside the pore with time for a decreasing contact angle. The operating conditions were as follows: TMP 0.9 bar, top wall velocity 0.9 m/s, and surface tension 0.019 N/m.

It was interesting to track the volume of the droplet in the feed channel and inside the pore as it changed with time. With the movement of the droplet over the surface, the volume in the channel changed slightly when the droplet encountered the pore opening. This was apparently because of the concave interface (toward the channel) that was formed at the pore opening. This volume was quite small and when it was normalized by the volume of the droplet, it represented only few percent and could be ignored, as depicted in Figure 14a. This figure shows the variations of the normalized volume of the droplet in the channel with time as the contact angle changed. As seen, the contact angle changed in increments of 2.5° in each cycle. When the droplet encountered the pore opening, a slight change in the volume was depicted as circled. When the contact angle became such that the critical pressure associated with the interface at the pore equaled the applied pressure, the droplet started to permeate. The droplet first permeated at a slower rate corresponding to the time at which the interface was advancing inside the pore. Once the interface broke through the exit of the pore opening, the permeation continued at a faster rate until the whole droplet had permeated. 

This is also depicted in Figure 14b, which shows the variations of the volume of the droplet inside the pore opening with time, normalized by the volume of the droplet. In this case, the scaled volume became more noticeable. As depicted in Figure 14b, several spikes appeared at the time when the droplet encountered the pore opening. Furthermore, the size of the spikes increased with the decrease in the contact angle. 

This implies that, as the contact angles increased, the interface inside the pore slightly flattened and the critical entry pressure decreased. As a result, the volume of that portion of the droplet inside the pore slightly increased until the contact angle reached a value that matched the critical pressure, at which time, the droplet started to permeate. In this case, the volume of the droplet inside the pore continued to increase until it totally filled the whole volume of the pore, it stayed constant for some time and then started to drop when the droplet in the channel had vanished. 

**Figure 15:**
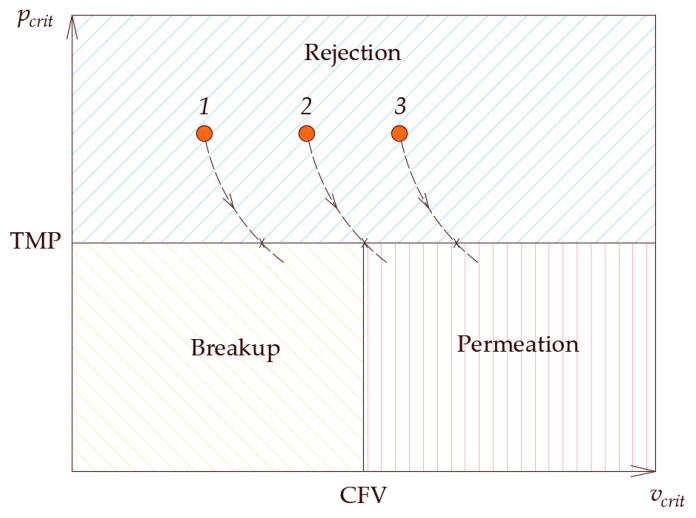
When the droplet is initially in state 1, the critical pressure is larger than the TMP (i.e., no permeation would occur) and the critical velocity of dislodgment is smaller than the CFV (i.e., rejection would occur). As the contact angle decreases, the critical entry pressure decreases and the critical velocity of dislodgment increases, as depicted by the dashed line. If the incremental decrease in the contact angle is small, the droplet will cross the constant TMP line, at which point, permeation starts, and if the critical velocity remains smaller than the CFV, breakup will occur. A similar pattern can be noticed for scenarios 2 and 3. In scenario 3, the droplet can either be pinned in the neighborhood of the TMP line or permeate.

To summarize the findings of this section, it is constructive to show the fate map on which the critical conditions and the operating conditions are plotted [51,52,53]. In Figure 15, the two-dimensional area is divided into three regions by the lines of constant operating conditions (i.e., the TMP and the CFV), while the critical conditions could assume any value inside the domain. If a droplet starts at point 1 (i.e., in the rejection region), $\mathrm{TMP}<p_{crit}$ and the droplet will not permeate; furthermore $\mathrm{CFV}>v_{crit}$ and the droplet will not be pinned. Now, with the decrease of the contact angle, the critical conditions change, i.e., $p_{crit}$ becomes smaller and $v_{crit}$ becomes larger. This will change the fates of the droplet as time passes once the critical conditions cross the TMP line. Therefore, for scenario 1, the droplet will continue to behave in a rejection manner until the critical pressure crosses the TMP line, in which case, the critical pressure becomes smaller than the TMP and the droplet starts to permeate. On the other hand, the critical velocity of dislodgment is still smaller than the CFV and the droplet will break up. For scenario 3, where the initial critical conditions are such that $\mathrm{TMP}<p_{crit}$ and $\mathrm{CFV}<v_{crit}$, the droplet will still experience rejection. As the contact angle continues to decrease and the critical conditions change, the droplets can experience different fates. As seen in Figure 15, the critical conditions for scenario 3 become such that $\mathrm{TMP}\geq p_{crit}$ and $\mathrm{CFV}<v_{crit}$; in this case the droplet experiences permeation rather than rejection. There are situations in which the increment change in the decrease of the contact angle is small and the droplet would experience pinning when the changes in the critical conditions get closer to the TMP line. In this case, the droplet experiences pinning, which is an interesting scenario depicted in the snapshots shown in Figure 12. In this case, if the decrease in the contact angle stops, the droplet will continue to be pinned at the entrance of the pore opening. This is a delicate condition as it represents the case in which the drag force by the crossflow field balances the interfacial tension force, as depicted in scenario 2. 

## 6. Conclusions

In this work, the effect of the contact angle on the behavior of the permeation process of an oil droplet at a membrane surface was studied in a microfiltration setup using CFD analysis. The microscopic study was conducted to highlight, at a small scale, the behavior of a single oil droplet upon encountering a pore opening for different contact angle scenarios. In this work, the wettability conditions at the surface of the membrane and inside the pore were the same. A formula was derived to determine the critical entry pressure when the wettability conditions were not the same. As a validation, this formula is shown to reduce to the equation derived when the contact angles are identical.

This study considered a domain with a rectangular cross-section that had a nonwetting affinity to the oil droplet. Therefore, when a droplet was placed at the surface, it assumed a spherical shape. The droplet was placed right along the centerline plane. A cylindrical hole in the middle of the domain represented a pore opening. The continuous fluid was sheared by the motion of the top wall and the established flow field stressed the droplet to force it to move along the surface. The contact angle at the droplet–water–membrane intersection (i.e., along the contact line) was initially 135° (i.e., nonwetting for the oil). Under these conditions, the droplet was displaced along the membrane by the crossflow field. When the droplet encountered the opening, it formed an interface. If the pressure across the cylindrical hole was smaller than the critical entry pressure, the droplet did not permeate and continued to move along the surface. 

In this work, we considered a droplet that was initially subject to a TMP that was smaller than the critical entry pressure and a CFV that was smaller than the critical velocity of dislodgment. Under these conditions, the droplet should not permeate and instead should get rejected, which was confirmed by the CFD analysis. When the contact angle decreases, the critical conditions also start to change. In particular, the critical entry pressure decreases (i.e., the droplet becomes more susceptible to permeation) and the critical velocity of dislodgment increases (i.e., the droplet becomes more susceptible to pinning). This will continue until the critical entry pressure becomes smaller than the TMP and the droplet starts to permeate, while the critical velocity of dislodgment continues to increase and the crossflow field will not have enough drag to dislodge the droplet, and therefore, permeation continues until the droplet fully passes through the pore. This was also confirmed via the CFD study. An interesting fate also emerged in this study when both the critical and operating conditions match. In this case, the TMP is not large enough to cause permeation and the CFV is also not large enough to cause detachment such that the droplet is pinned at the pore opening. This was also confirmed via the conducted CFD study. 

## Figures and Tables

**Figure A1:**
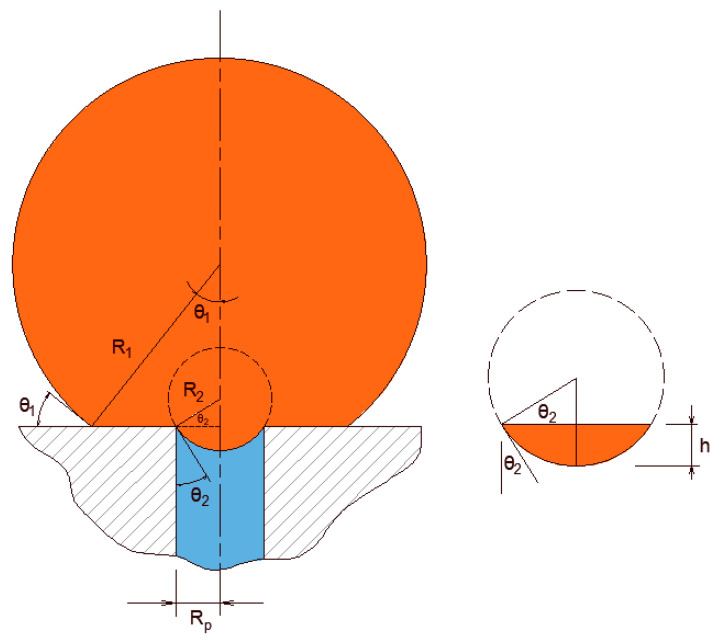
An oil droplet at the outcrop of a pore opening forms two interfaces. The critical entry pressure is achieved when the contact angle at the pore opening is the static contact angle.

## Data Availability

Not applicable.

## Appendix A

In this section, a derivation is given for the critical entry pressure of a droplet that is pinned over a pore opening when the contact angles at the surface and in the pore are different. The start of the permeation of an oil droplet that is pinned at a pore opening is marked by the moment when the interface formed at the entrance of the pore assumes the static contact angle. In produced water applications, oil droplets are typically of sizes on the order of tens of microns or less, which suggests that the droplet may be considered spherical in shape. In this case, volume calculations can be used to determine the radii of curvature of the two interfaces (Figure A1). When the contact angle of the interface at the pore opening assumes the static contact angle, the critical entry pressure can be calculated using:

(A1) $$p_{crit}=p_{1}-p_{3}=\left(p_{2}-p_{3}\right)-\left(p_{2}-p_{1}\right),$$

where $p_{1}$ is the pressure in the feed channel (i.e., outside the droplet), $p_{2}$ is the pressure inside the droplet, and $p_{3}$ is the pressure at the exit of the pore.

(A2) $$p_{2}-p_{1}=\frac{2\sigma }{R_{1}},$$

(A3) $$p_{2}-p_{3}=\frac{2\sigma }{R_{2}},$$

where $R_{1}$ and $R_{2}$ are the radii of the two interfaces (i.e., in the feed channel and inside the pore). Substitution into Equation (A1) yields:

(A4) $$p_{crit}=\frac{2\sigma }{R_{2}}-\frac{2\sigma }{R_{1}}.$$

To simplify calculations, the radii of curvature of the two interfaces, i.e., $R_{1}$ and $R_{2}$, need to be calculated in terms of the radius of the pore and the radius of the droplet. The radius of curvature of the interface inside the pore can easily be calculated in terms of the radius of the pore:

(A5) $$R_{2}=R_{p}/\cos\vartheta_{2}.$$

However, the radius of curvature of the interface at the surface is not readily available and requires calculating the two volumes comprising the droplet (i.e., inside the pore and at the surface), which are calculated as follows.

The volume of a spherical segment may be determined using:

(A6) $$V=\overset{h}{\underset{0}{\int }}\pi r^{2}dy,$$

where *h* is the height of the spherical segment. Evaluating the volume of that portion of the droplet inside the pore opening is done as follows:

(A7) $$V_{2}=\frac{\pi }{3}h_{2}^{2}\left(3R_{2}-h_{2}\right),$$

(A8) $$h_{2}=R_{2}\left(1-\sin\vartheta_{2}\right)=\frac{1-\sin\vartheta_{2}}{\cos\vartheta_{2}}R_{p},$$

(A9) $$V_{2}=\frac{\pi }{3}\frac{{\left(1-\sin\vartheta_{2}\right)}^{2}}{\cos^{3}\vartheta_{2}}R_{p}^{3}\left(2+\sin\vartheta_{2}\right)$$

(A10) $$=\frac{\pi }{3}\frac{\left(2-3\sin\vartheta_{2}+\sin^{3}\vartheta_{2}\right)}{\cos^{3}\vartheta_{2}}R_{p}^{3}.$$

Similarly, the volume of that portion of the droplet at the surface is calculated using:

(A11) $$V_{1}=\frac{4}{3}\pi R_{1}^{3}-\frac{\pi }{3}h_{1}^{2}\left(3R_{1}-h_{1}\right),$$

(A12) $$h_{1}=R_{1}\left(1-\cos\vartheta_{1}\right),$$

(A13) $$V_{1}=\frac{4}{3}\pi R_{1}^{3}-\frac{\pi }{3}R_{1}^{3}{\left(1-\cos\vartheta_{1}\right)}^{2}\left(2+\cos\vartheta_{1}\right)$$

(A14) $$=\frac{\pi }{3}R_{1}^{3}\left(2+3\cos\vartheta_{1}-\cos^{3}\vartheta_{1}\right),$$

(A15) $$V_{1}=V_{d}-V_{2},$$

(A16) $$\frac{\pi }{3}R_{1}^{3}\left(2+3\cos\vartheta_{1}-\cos^{3}\vartheta_{1}\right)=\frac{4}{3}\pi R_{d}^{3}-\frac{\pi }{3}\frac{\left(2-3\sin\vartheta_{2}+\sin^{3}\vartheta_{2}\right)}{\cos^{3}\vartheta_{2}}R_{p}^{3},$$

(A17) $$R_{1}^{3}\left(2+3\cos\vartheta_{1}-\cos^{3}\vartheta_{1}\right)=4R_{d}^{3}-\frac{\left(2-3\sin\vartheta_{2}+\sin^{3}\vartheta_{2}\right)}{\cos^{3}\vartheta_{2}}R_{p}^{3},$$

(A18) $$R_{1}=\frac{R_{p}}{\cos\vartheta_{2}}\sqrt[{3}]{\frac{4{\left(\frac{R_{d}}{R_{p}}\right)}^{3}\cos^{3}\vartheta_{2}-\left(2-3\sin\vartheta_{2}+\sin^{3}\vartheta_{2}\right)}{\left(2+3\cos\vartheta_{1}-\cos^{3}\vartheta_{1}\right)}}.$$

Using Equation (A4):

(A19) $$p_{crit}=\frac{2\sigma \cos\vartheta_{2}}{R_{P}}\left[1-\sqrt[{3}]{\frac{\left(2+3\cos\vartheta_{1}-\cos^{3}\vartheta_{1}\right)}{4{\left(\frac{R_{d}}{R_{p}}\right)}^{3}\cos^{3}\vartheta_{2}-\left(2-3\sin\vartheta_{2}+\sin^{3}\vartheta_{2}\right)}}\right]\;.$$

The variations in the entry pressure with the ratio of the droplet diameter to the pore diameter are shown in Figure A2 for different values of the contact angle of the interface at the entrance of the pore opening (at a fixed value of the contact angle at the horizontal surface of 150°). From this figure, when the contact angles of the two interfaces at the pore opening and the surface are the same, the normalized entry pressure is smaller for smaller droplets. When the contact angle at the pore entrance decreases, the entry pressure decreases further.
